# Special Issue, “Viruses and Eye Diseases: Emerging Mechanisms, Host–Virus Interactions, and Future Directions”

**DOI:** 10.3390/v18090990

**Published:** 2026-09-09

**Authors:** Deepak Shukla

**Affiliations:** 1 Department of Ophthalmology and Visual Sciences, College of Medicine, University of Illinois Chicago, Chicago, IL 60612, USA; dshukla@uic.edu; 2 Department of Microbiology and Immunology, College of Medicine, University of Illinois Chicago, Chicago, IL 60612, USA

## 1. Introduction

Vision-threatening viral infections remain a major cause of ocular morbidity and blindness worldwide despite substantial advances in antiviral therapy, molecular virology, and ocular immunology. Herpes simplex virus type 1 (HSV-1), herpes simplex virus type 2 (HSV-2), and cytomegalovirus (CMV) continue to impose a significant clinical burden through corneal disease, retinal infections, recurrent inflammation, and permanent visual impairment, particularly in immunocompromised individuals and neonates. Although considerable progress has been made in understanding viral replication and pathogenesis, many critical questions remain unanswered regarding the complex interplay between viruses and host tissues that ultimately determines disease severity, persistence, immune-mediated damage, and long-term visual outcomes. Increasing evidence suggests that ocular viral diseases cannot be explained solely by viral replication. Rather, disease reflects a dynamic interaction between viral factors, host immune responses, extracellular matrix remodeling, and tissue repair pathways. Furthermore, recent discoveries indicate that viral infections may contribute to chronic retinal degeneration and age-related ocular disorders beyond their traditionally recognized acute manifestations. These evolving concepts highlight the need for multidisciplinary approaches integrating virology, immunology, cell biology, neuroscience, and translational medicine.

This Special Issue, “Viruses and Eye Diseases”, brings together three original research articles and two authoritative reviews that collectively capture several emerging themes currently shaping the field. Together, these contributions span multiple herpesviruses, different ocular tissues, innate and adaptive immune responses, extracellular matrix biology, viral latency, and retinal degeneration. Importantly, they emphasize that host-directed mechanisms are becoming increasingly central to understanding viral pathogenesis and identifying new therapeutic opportunities.

## 2. Contributions

One recurring theme across this Special Issue is the importance of regulated host immune responses. Carter and colleagues [1] investigated canonical pyroptosis during productive replication of murine and human cytomegaloviruses and demonstrated striking differences between MCMV and HCMV in their ability to induce inflammasome-associated pathways. Their work suggests that pyroptosis is not a universal feature of CMV infection but instead depends on both viral species and host cell type. These findings have important implications for interpreting animal models of CMV retinitis and underscore the need to carefully distinguish conserved from species-specific mechanisms when translating discoveries from murine systems to human disease.

A second major theme emerging from this Special Issue is the expanding role of extracellular matrix remodeling in viral infection. Hopkins and colleagues [2] identify heparanase 2 (HPSE2) as a previously underappreciated regulator of HSV-2 replication. By modulating the activity of the extracellular matrix enzyme heparanase (HPSE), its isoform, HPSE2, influences both viral entry and egress by regulating cell-surface heparan sulfate. These findings further strengthen the concept that host glycocalyx remodeling represents an attractive therapeutic target that is less susceptible to the development of antiviral resistance than conventional virus-directed approaches. More broadly, this work highlights the growing recognition of the importance of extracellular matrix biology in viral pathogenesis.

The third original investigation addresses one of the defining biological properties of HSV infection, that is, latency. Jaggi and Ghiasi [3] demonstrate that CD80, a key costimulatory molecule, significantly influences HSV-1 latency and inflammatory responses without altering viral reactivation. Their findings reveal an intriguing dissociation between latency establishment and subsequent reactivation, suggesting that these processes are regulated by distinct molecular pathways. The study further illustrates the complexity of immune regulation during HSV infection and raises the possibility that selective modulation of immune costimulatory pathways may reduce immunopathology while preserving essential antiviral defense.

Complementing these mechanistic studies are two timely review articles that broaden the scope of this Special Issue. Ayala-Peña [4] synthesizes growing evidence linking HSV-1 infection of retinal pigment epithelial cells with age-related macular degeneration (AMD), highlighting potential interactions between viral infection, amyloid-β accumulation, chronic inflammation, and retinal degeneration. This emerging area challenges the traditional view of HSV-1 as solely an acute ocular pathogen and raises the provocative possibility that viral infection may contribute to chronic neurodegenerative processes within the retina. Although direct causal relationships remain to be established, this hypothesis opens exciting avenues for future investigation into the infectious contributors to age-related ocular disease.

Ghiasi’s perspective provides an insightful historical account of the discovery and evolution of the biology of latency-associated transcripts (LATs) [5]. Beyond documenting scientific chronology, the article illustrates how incremental discoveries, vigorous scientific debate, and technological advances collectively shaped our current understanding of HSV latency, apoptosis, immune evasion, and viral reactivation. This historical perspective serves as a valuable reminder that scientific progress often emerges through continuous refinement of ideas rather than isolated breakthroughs.

Collectively, the articles in this Special Issue reveal several important trends. First, host determinants, including immune signaling pathways, extracellular matrix remodeling, and cellular stress responses, are increasingly recognized as major regulators of ocular viral disease. Second, viral pathogenesis extends beyond direct cytopathic effects and encompasses long-term consequences such as chronic inflammation, tissue remodeling, and potentially neurodegenerative processes. Third, advances in molecular genetics and experimental models continue to uncover previously unrecognized mechanisms that may ultimately translate into innovative therapeutic strategies.

## 3. Conclusions and Outlook

Despite these advances, substantial knowledge gaps remain in the field of viruses and the eye. The precise molecular events governing the transition between productive viral infection, persistence, latency, and reactivation remain incompletely understood. The mechanisms by which viruses interact with resident ocular immune cells and neural tissues require further investigation, particularly through multi-omics approaches, including single-cell and spatial transcriptomics. Similarly, the contribution of extracellular matrix dynamics, mechanobiology, and tissue repair pathways to viral dissemination and immune regulation remains only partially explored. Better understanding of these processes may reveal opportunities for host-directed therapies capable of limiting disease while reducing the emergence of antiviral resistance.

Future research should also prioritize the development of physiologically relevant experimental systems, including organoids, human retinal and corneal models, advanced imaging technologies, and integrated multi-omics approaches. Artificial intelligence-assisted image analysis, systems biology, and computational modeling will likely accelerate the identification of predictive biomarkers and therapeutic targets. At the translational level, combining direct-acting antivirals with host-targeted therapies that modulate inflammation, oxidative stress, extracellular matrix remodeling, or immune regulation may provide more durable and effective treatments than antiviral monotherapy alone. Finally, growing appreciation of the connections between viral infection and chronic ocular degeneration underscores the importance of interdisciplinary collaboration among virologists, ophthalmologists, immunologists, neuroscientists, glycobiologists, and bioengineers. Such collaborative efforts will be essential for translating mechanistic discoveries into improved diagnostics, preventive strategies, and therapies that preserve vision.
